# Accurate characterization of β-amyloid (Aβ40, Aβ42) standards using species-specific isotope dilution by means of HPLC-ICP-MS/MS

**DOI:** 10.1007/s00216-021-03571-6

**Published:** 2021-08-06

**Authors:** Martin Schaier, Gerrit Hermann, Gunda Koellensperger, Sarah Theiner

**Affiliations:** 1 Institute of Analytical Chemistry, Faculty of Chemistry, University of Vienna, Waehringer Strasse 38, 1090 Vienna, Austria; 2 Institute of Analytical Chemistry, Department of Chemistry, University of Natural Resources and Life Sciences, Muthgasse 18, 1190 Vienna, Austria; 3 ISOtopic solutions e.U., Waehringer Strasse 38, 1090 Vienna, Austria

**Keywords:** ICP-MS, Species-specific isotope dilution, Traceability, Amyloid beta peptide, Alzheimer’s disease, Speciation analysis

## Abstract

The amyloid β peptide, as one of the main components in senile plaque, represents a defining pathological feature for Alzheimer’s disease, and is therefore commonly used as a biomarker for this disease in clinical analysis. However, the selection of suitable standards is limited here, since only a few are commercially available, and these suffer from varying purity. Hence, the accurate characterization of these standards is of great importance. In this study, we developed a method for the traceable quantification of the peptide content using species-specific isotope dilution and ICP-MS/MS detection. It is based on the separation of the sulfur-containing amino acids methionine and cysteine after oxidation and hydrolysis of the peptide. Using a strong anion exchange column, both amino acids could be separated from each other, as well as from their oxidized forms and sulfate. The sulfur content was determined via ICP-MS/MS using oxygen as reaction gas. Species-specific isotope dilution was enabled by using a ^34^S-labeled yeast hydrolysate, containing methionine sulfone and cysteic acid with different isotopic composition. The peptide contents of Aβ standards (Aβ40,42), as well as myoglobin and lysozyme with different degrees of purity, were determined. For validation purposes, the standard reference material NIST 2389a, which contains the amino acids in a similar concentration, was subjected to the developed sample preparation and analysis method. In addition to accounting for errors during sample preparation, high levels of accuracy and precision could be obtained using this method, making it fit-for-purpose for the characterization of peptide standards.

**Graphical abstract:**

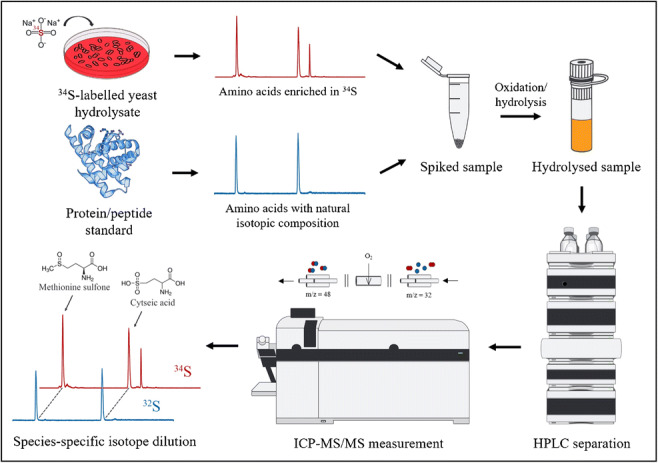

**Supplementary Information:**

The online version contains supplementary material available at 10.1007/s00216-021-03571-6.

## Introduction

Due to the steady increase in global life expectancy [1], age-related diseases such as Alzheimer’s disease (AD), the most common form of neurodegenerative disorder [2], are becoming a growing problem. According to reports from the Word Health Organization in 2018 [3], the number of deaths due to dementias has more than doubled since 2000, making it the 5^th^ leading cause of death worldwide. AD is characterized by a gradual decline in different cognitive domains such as memory, language, or personality [4], which makes it difficult to diagnose in early stages, as these symptoms are the result of years of neurological damage [5]. Because of this impairment in basic body functions, death is ultimately caused by the vulnerability of patients to infections like pneumonia [6].

The most defining pathological features of this disease are neurofibrillary tangles and senile plaque in the hippocampus of the brain [7]. As a result, there has been great interest in research for the main component of this plaque, the β-amyloid peptide (Aβ), which consists of 38–42 amino acids and originates from the amyloid precursor protein (APP) [8]. APP, which is a transmembrane protein abundant in the central nervous system [9], is believed to be involved in cell growth and health, but the exact physiological function is not yet fully understood [10]. If excessive amounts of the Aβ peptide are produced (as in the case of AD), its normal functions are inhibited through aggregation and toxic oligomers are formed, which are able to cause oxidative damage to the brain and then accumulate as senile plaque [9–11].

Therefore, Aβ is widely used as biomarker for Alzheimer’s disease, alongside neurogranin, phosphorylated tau, and total tau [12]. Specifically, the ratio between the most abundant fragments Aβ40 and Aβ42 is measured in cerebrospinal fluid (CSF). While the concentration of Aβ42 changes significantly with AD (decrease because of Aβ aggregation), Aβ40 remains relatively constant in most patients, thus providing normalization for measurements [13]. Aβ40 is more abundant with reported levels of about 2–20 ng mL^−1^ in CSF compared to 0.2–1 ng mL^−1^ of Aβ42, which can decrease in AD patients by up to 50% [14–17]. The most important tools for quantitative measurements in CSF are ELISA assays, which employ multiple antibodies with high affinity to Aβ peptides [14, 15]. In addition, amyloid deposits in the brain are visualized with positron emission tomography (PET) scans using ^18^F-fluorodeoxyglucose, since they showed good accordance to the senile plaque found in autopsy [18].

Due to the high level of responsibility that goes with patient samples, high accuracy and specificity are basic requirements for clinical analysis [19]. Even small differences in concentration can be decisive for the final diagnosis, which is why it is important to ensure traceability of the measurements, as otherwise results are dependent on the laboratory and procedure used [19, 20]. According to the international terminology of metrology (JCGM 200:2012, Clause 2.41), traceability is defined by a “documented unbroken chain of calibrations, each contributing to the measurement uncertainty” [21]. In order to ensure equivalence of results between laboratories (ISO 17511:2020) for in vitro diagnostics, a calibration hierarchy must be established, where each measurement result can be traced back to the calibration carried out previously [22]. Measurement units, standards, or procedures can serve as a reference. In the ideal case, a primary reference measurement procedure is used in which the quantity value can be traced back to the basic units of the International System (SI) [23]. Since this is not possible for every analyte, the use of certified reference materials and reference measurements, which are accompanied by measurement uncertainties and traceability provided by an authoritative body, is more common [21, 23]. However, due to their limited availability, particularly in the area of biological standards such as proteins, manufacturers often have to resort to in-house calibration procedures [24].

Typically, protein assays based on UV absorption, Biuret methods, colorimetric dye, or fluorescence are used for characterization. Accordingly, the measurements are usually carried out in a non-traceable manner without internal standard and with poor accuracy [20]. One commonly used method is the colorimetric Bradford assay where concentrations are obtained using a species-unspecific calibrant such as albumin [25]. A better approach for the quantification of proteins and peptides is amino acid analysis using ion exchange or reversed-phase HPLC, which, however, is usually limited by UV detection in terms of accuracy, selectivity, and sensitivity [26, 27].

These limitations can be overcome by the use of amino acid–based isotope dilution liquid chromatography mass spectrometry (ID-LC-MS) for the certification of protein reference materials. The method employs either isotope-labeled (^13^C or ^15^N) amino acids, peptides, or proteins as internal standard, mainly to account for variations in the yield of the analytes from biological samples, where complex matrices can interfere with the measurement. For accurate quantification of the protein, a complete hydrolysis of the protein into the individual amino acids is required. In this regard, Aβ peptides were for example quantified in CSF from Alzheimer’s disease patients by immunoaffinity purification and ID-LC-MS analysis [28]. In another instance, ^13^C-labeled Aβ peptides were used as internal standard for the quantification in human plasma using MALDI-TOF [29].

Another MS-based approach for protein quantification makes use of the advantages of inductively coupled plasma-mass spectrometry (ICP-MS) for highly sensitive element quantification. Since the majority of proteins contain sulfur via the amino acids cysteine and methionine or are phosphorylated [30, 31], proteins or peptides are hydrolyzed, and an amino acid analysis based on the sulfur/phosphor content is carried out using HPLC-ICP-MS [20]. The measurement of sulfur or phosphor allows the direct traceability to SI units [23].

Because of its capabilities to measure isotopic compositions, ICP-MS also allows the use of isotope dilution analysis (IDA), which offers high degrees of accuracy and precision for quantitative measurements and SI unit traceability [23, 32]. A recent study by Feng et al. [33] showed successful results for peptide quantification using species-unspecific isotope dilution, employing an inorganic ^34^S spike, which was added on-line after the column. This approach is versatile and fast, reducing sample preparation significantly and preventing precipitation. However, it is only able to correct instrumental errors during measurement.

Ideally, species-specific isotope dilution is used, with an isotopically labeled form of the examined species being added during sample preparation [34]. With this approach, systematic errors, sample loss, and transformation of the species can also be accounted for [35]. However, it must be considered that this type of isotope dilution is often limited by the availability of isotopically enriched standards. Therefore, independent synthesis is usually performed, which can be done either by adding labeled substrates to specific cell cultures and thus producing the required molecules (in vivo labeling) or via multi-step chemical synthesis [36, 37]. Both methods require complex procedures, often achieving only low yields, which is why method development has become crucial in this area.

In this work, we developed a traceable method for the characterization of protein and peptide standards using species-specific isotope dilution by means of HPLC-ICP-MS/MS. It is an extension of the quantification strategy implemented in recent works [20, 38]. For this purpose, the proteins were subjected to acid hydrolysis and oxidized in order to obtain the sulfur-containing amino acids cysteic acid and methionine sulfone, which were then separated using a strong anion exchange column and used to determine the peptide content. By employing a ^34^S-labeled yeast hydrolysate, errors during sample preparation and measurement could be successfully removed, leading to accurate and reproducible results.

## Experimental

### Chemicals and reagents

Ultrapure water (18.2 MΩ cm, ELGA Water Purification System, Purelab Ultra MK 2, UK or 18.2 MΩ cm, Milli-Q Advantage, Darmstadt, Germany) and nitric acid (≥ 69%, Rotipuran Supra, Carl Roth, Karlsruhe, Germany) were used in dilutions for ICP-MS measurements. The sulfur standard solution (1000 mg L^−1^, H_2_SO_4_ in 2% HNO_3_) was purchased from Labkings (Hilversum, The Netherlands). The ^34^S-enriched NaSO_4_ with 99.89% isotopic content (^34^S) and 98% chemical purity was purchased from Eurisotop (Saint-Aubin Cedex, France). The standards for lysozyme (from chicken egg white) with purities of > 90% and > 99% (VETRANAL^TM^) and myoglobin with purities of 95–100% and > 98% (SDS-PAGE), as well as the amino acid calibrants, namely methionine, methionine sulfone, cysteine, and cysteic acid, were all purchased by Sigma-Aldrich (Steinheim, Germany). The synthetic β-amyloid standards β-Amyloid 1–40 (Ultra Pure, TFA) and β-Amyloid 1–42 (Ultra Pure, TFA) were purchased from r-Peptide (Georgia, USA). ^34^S-labeled yeast hydrolysates were provided by ISOtopic solutions (Vienna, Austria). For validation of the analytical method, the NIST standard reference material SRM 2389a, amino acids in 0.1 mol L^−1^ hydrochloric acid (National Institute of Standards and Technology, Gaithersburg, MD, USA) was used. The ammonium acetate buffer applied as eluent for chromatography was derived from ammonium hydroxide solution (25%, Suprapur) and acetic acid (100%, Suprapur), which were purchased from Lactan (Graz, Austria).

### Sample preparation

#### Hydrolysis/oxidation of the proteins

β-Amyloid (around 1 mg, corresponding to the vial content) was mixed with 1 mL 1% NH_4_OH. To dissolve the entire content, the solution was added via a syringe, which penetrates the membrane of the vial. After that, a homogenization step was done by sonication for 1 min. The solution was then aliquoted in 100-μL fractions and 3 aliquots of each peptide (Aβ40, Aβ42) were used, while the others were stored at 4 °C. This procedure was necessary because the peptides could not be weighed in due to electrostatic interactions inside of the vial. Myoglobin and lysozyme (10 mg of each protein) were dissolved in 2 mL of 0.9% NaCl, and 3 aliquots were taken (each 40 μL). For NIST 2389a, four aliquots of the solution (each 10 μL) were directly weighed in. Each of those fractions was spiked with 20 μL of the ^34^S-enriched yeast hydrolysate. Subsequently, 500 μL of performic acid was added and the samples were incubated for 15 min at 65 °C. After that, they were evaporated to dryness using a GeneVac EZ-2 centrifugal evaporator (SP Industries Inc., Warminister, USA), mixed with 1 mL of 6 M HCl and incubated again for 24 h at 100 °C. The now oxidized and hydrolyzed samples were vortexed and centrifuged at 10,000 rcf for 5 min with a Hermle Z 446K centrifuge (HERMLE Labortechnik GmbH, Wehingen, Germany). The samples were transferred to Eppendorf tubes, diluted 1:10 with the buffer (25 mM CH_3_COONH_4_) in HPLC vials, and were directly used for analysis.

#### Reduction of the sulfur background

In order to minimize sulfur contaminations, sample preparation was carried out in an ISO class 8 clean room, with measurements being done in ISO class 7. All consumables were subjected to a cleaning procedure. They were first treated with 10% HNO_3_ for 24 h, then with 1% HNO_3_ for the same time and finally rinsed with ultrapure water. After drying in a fume hood, the consumables were stored in plastic bags. The HNO_3_ was always replaced after multiple cleaning cycles. Either polypropylene (PP) or perfluoroalkoxy alkane (PFA) labware were used because glass showed a higher sulfate background.

To keep the sulfate contamination at a low level during the measurements, the column was cleaned on a regular basis. For this purpose, the column was first rinsed with ultrapure water for several minutes, then with 200 mM HCl in 80% ACN for 1 h, subsequently again with water for 1–2 h and finally with the eluent for 30 min. This reduced the sulfate background by approximately 92%.

#### Species-specific isotope dilution

**Fig. 1:**
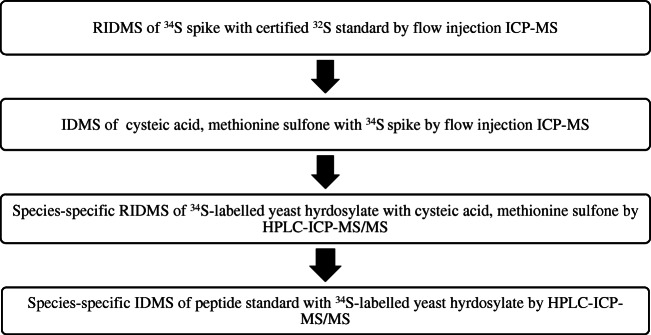
Overview of the measurements necessary for traceable quantifications using species-specific isotope dilution

In order to ensure traceability with this type of isotope dilution, each standard had to be quantified step by step. Therefore, first, the ^34^S spike had to be measured precisely using reverse isotope dilution. The term reverse here refers to the characterization of the spike instead of the sample. After that, the two standards of the oxidized amino acids (methionine sulfone, cysteic acid) were characterized using this spike. By adding these standards to the ^34^S-labeled yeast hydrolysate, its composition could be characterized using the anion exchange column. Figure 1 provides an overview of the measurements required.

### Instrumentation

#### ICP-MS/MS

An Agilent 8800 ICP-MS/MS instrument (Agilent Technologies, Tokyo, Japan) with oxygen as reaction gas was used to determine the sulfur content. The ICP-MS parameters were tuned on a daily base to achieve high sensitivity. The ICP-MS was equipped with a MicroMist nebulizer with a sample uptake rate of ∼ 0.25 mL min^−1^ and standard nickel cones. All sulfur isotopes were measured using oxygen as reaction gas (flow rate = 0.32 mL min^−1^) with mass selection steps on both quadrupoles. The following isotopes were monitored with a respective integration time of 0.1 s: ^32^S^16^O^+^, ^33^S^16^O^+^, ^34^S^16^O^+^, and ^36^S^16^O^+^. The instrument was coupled to an Agilent 1260 Infinity Bio-Inert HPLC system (Agilent Technologies, Waldbronn, Germany). The Agilent MassHunter software package (Workstation Software, Version C.01.03, 2016) was used for data evaluation. The instrumental parameters for the ICP-MS measurements are summarized in Table 1.

**Table 1:** Instrumental parameters for the ICP-MS/MS measurements

RF power [W]	1550
Nebulizer	MicroMist
Spray chamber	Scott double-pass
Spray chamber temp. [°C]	2
Cone materials	Ni
Plasma gas flow (Ar) [L min^−1^]	15.0
Auxiliary gas flow (Ar) [L min^−1^]	1.10
Nebulizer gas flow (Ar) [L min^−1^]	0.90
Reaction gas flow (O_2_) [mL min^−1^]	0.32
Monitored isotopes	^32^S^16^O, ^33^S^16^O, ^34^S^16^O, ^36^S^16^O
Integration time	0.1 s

#### HPLC system

The final separation of the sulfur-containing amino acids was carried out with a Thermo Scientific Dionex^TM^ IonPac^TM^ AS22 anion exchange column (6 μm, 2 × 250 mm, Thermo Scientific Inc., MA, USA). In preliminary experiments, other columns with reversed-phase chemistry, specifically Atlantis T3 (3 μm, 4.6 × 150 mm, Waters), were tested, providing insufficient separation of the amino acids. The summarized HPLC parameters are shown in Table 2.

**Table 2:** HPLC conditions for the different columns used in the separation

Column	Atlantis T3		Dionex^TM^ IonPac^TM^ AS22	
Flow rate [μL min^−1^]	100		400	
Injection volume [μL]	10		10	
Buffer A	10 mM CH_3_COONH_4_, pH = 5		20 mM CH_3_COONH_4_, pH = 8.0	
Buffer B	MeOH		400 mM CH_3_COONH_4_, pH = 8.0	
Gradient	0–1 min	100% A	0–4 min	100% A
	1–5.5 min	Ramp to 20% B, 80% A	4–7 min	Ramp to 100% B
	5.5–6 min	20% B, 80% A	7–12 min	100% B
	6–6.05 min	Ramp to 100% A	12–12.1 min	Ramp to 100% A
	6.05–7 min	100% A	12.1–16 min	100% A

## Results and discussion

### Development of the amino acid separation

Reversed-phase and anion exchange chromatography were evaluated for the separation of the amino acids cysteine and methionine, their oxidized forms cysteic acid and methionine sulfone, and sulfate, according to results in the literature [20, 39]. In addition to the quantification of the peptides, optimal separation enabled to evaluate if the oxidation in the sample preparation was complete.

Using gradient elution, the separation of cysteine and methionine was possible using reversed-phase chromatography; however, they showed an overlap with their oxidized forms (see Supplementary information (ESM) Fig. S1). In addition, evaluation of cysteine was difficult due to the similar retention time of sulfate.

**Fig. 2:**
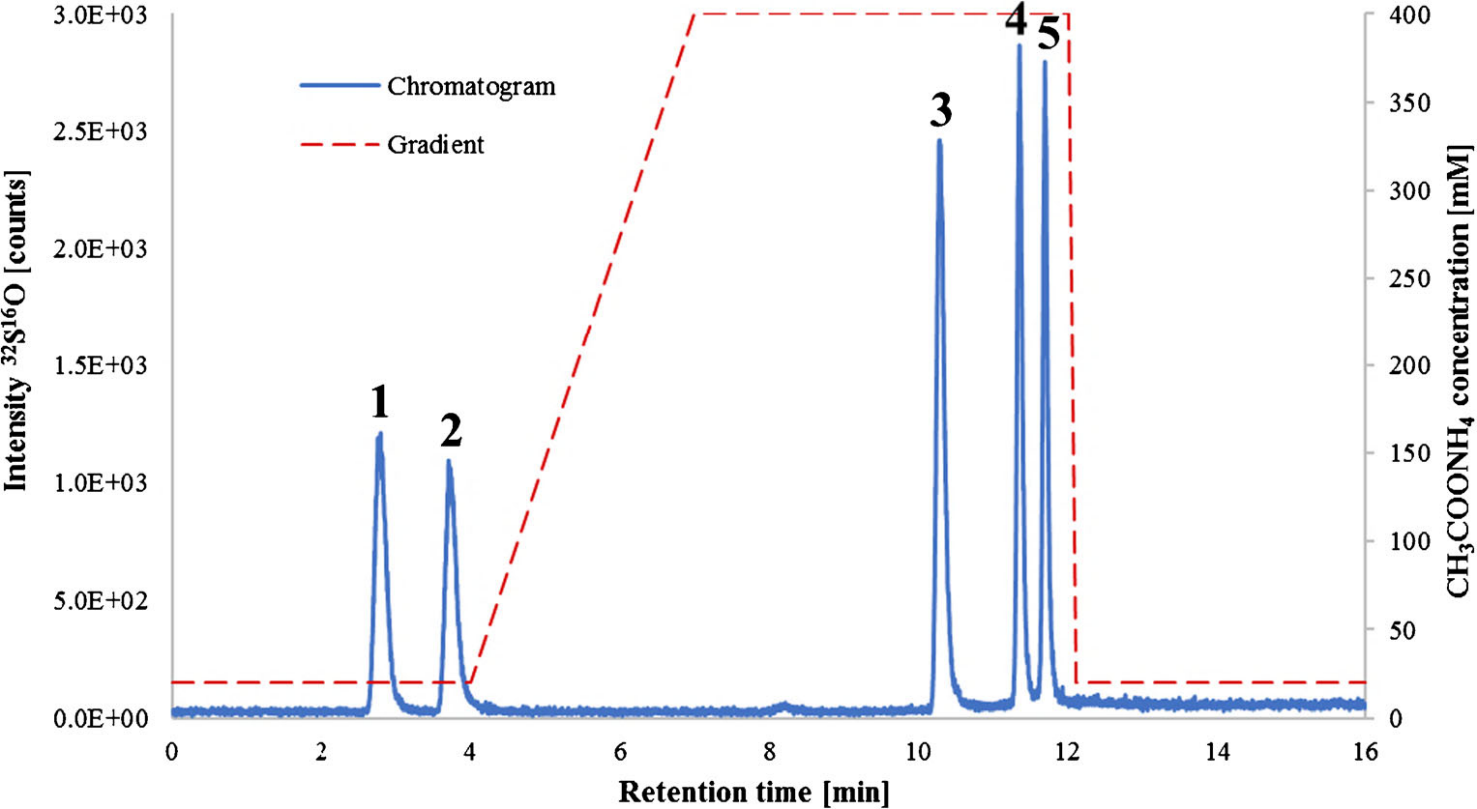
Chromatogram of an amino acid mixture containing methionine, cysteine, and their oxidized forms in addition to sulfate. The concentrations were selected so that each substance contained approximately 100 μg L^−1^ sulfur. The sulfur trace was monitored using HPLC-ICP-MS/MS with oxygen as reaction gas. The optimized gradient is seen in the background to better illustrate the isocratic steps. The substances eluted in the following order: (1) methionine, (2) methionine sulfone, (3) cysteine, (4) cysteic acid, and (5) sulfate

Therefore, anion exchange chromatography was used as an alternative. First isocratic elution was tested at different CH_3_COONH_4_ concentrations to evaluate the retention time of the respective substances. Methionine and methionine sulfone eluted at 20 mM CH_3_COONH_4_, cysteine and cysteic acid at 100 mM CH_3_COONH_4_, and sulfate required 400 mM CH_3_COONH_4_ for an acceptable retention time. Based on these results, a gradient was developed to ensure that the substances elute within the isocratic steps, as the sulfur background increases with higher salt concentration and this could have an impact on the isotope dilution (ID) measurements. The gradient started with isocratic elution at a low concentration of CH_3_COONH_4_ to achieve optimal separation of methionine and methionine sulfone within 4 min. Directly after the methionine sulfone peak, the concentration was rapidly increased (400 mM CH_3_COONH_4_) to elute the remaining substances as quickly as possible. The increase was gradual over 3 min to ensure a stable eluent for the next isocratic step. Due to cysteine having more negatively charged ions than methionine in a basic environment, it eluted later at 10.3 min. Cysteic acid and sulfate have even higher acidity and thus showed the longest retention on the column with 11.3 and 11.7 min. After the elution of all the analytes, the gradient was changed back to the starting conditions (20 mM CH_3_COONH_4_) and left for an additional 4 min to equilibrate the system for the next measurement. This made it possible to reliably separate the 5 compounds within a measurement time of 16 min, as can be seen in Fig. 2.

### Separation of the protein/peptide standards

**Fig. 3:**
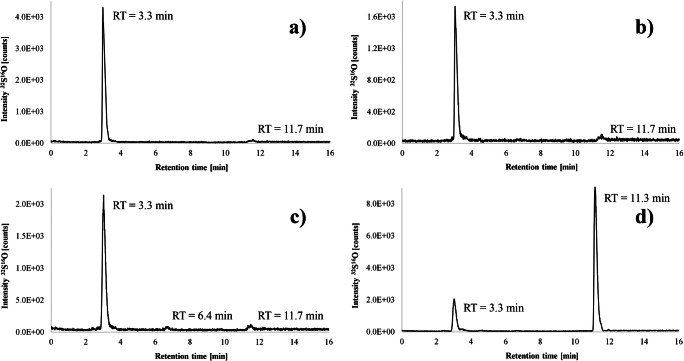
Chromatograms of the peptide standards after being exposed to hydrolysis and oxidation. The sulfur traces of Aβ40 (**a**), Aβ42 (**b**), myoglobin (**c**), and lysozyme (**d**) were monitored by HPLC-ICP-MS/MS with oxygen as reaction gas. Prior to analysis, the samples were further diluted 1:10, resulting in substance concentrations of approximately 10 mg L^−1^ for the Aβ peptides and 20 mg L^−1^ for the proteins

Using the optimized gradient, the hydrolyzed protein and peptide standards were separated by anion exchange chromatography and ICP-MS/MS detection. In addition to Aβ40 and Aβ42, the proteins myoglobin and lysozyme were measured. For Aβ40, only one peak at 3.3 min was visible (Fig. 3a), which corresponds to methionine sulfone. The amino acid thus appears to have been almost completely oxidized, proving the sample preparation to be successful. A slight increase in intensity is only noticeable at 11.7 min because of the low sulfate background. Aβ42 showed an identical pattern (Fig. 3b), but the area of methionine sulfone was significantly lower despite the same concentration, leading to more noise in the chromatogram.

The chromatogram of myoglobin resembled the one of the Aβ peptides, containing only 2 methionine molecules in its sequence (Fig. 3c). Due to the small peak at 6.4 min, it can be assumed that a slight contamination with cysteine may have occurred. Lysozyme, which contains 2 methionine and 8 cysteine molecules, showed a comparatively higher sensitivity with two peaks visible at 3.3 and 11.3 min for their respective oxidized forms (Fig. 3d). The ratio of the peak areas was in good agreement with the number of sulfur-containing amino acids.

### Quantification of the peptide content by species-specific isotope dilution

Using the optimized gradient, calibration curves of the individual standards were created in a concentration range of 50–1000 μg L^−1^ (ESM Fig. S2). Limits of quantification (LOQ) were determined by means of a blank (0.6 M HCl), calculated as the mean value of 10 measurements of the blank +10 times the standard deviation of the blank measurements. For the LOQ of sulfate, the peak areas of the blank were used. However, since no peaks were visible for the amino acids, the counts around their retention times had to be employed. Accordingly, cysteic acid showed a higher LOQ because of an elevated sulfur background at 400 mM CH_3_COONH_4_ (Table 3).

**Table 3:** Retention times and limits of quantification for the measured substances using the optimized HPLC-ICP-MS/MS method. The LOQ values were also given as sulfur concentration in order to better reflect the limitations of HPLC-ICP-MS/MS

Substance	Retention time [min]	LOQ substance [μM]	LOQ sulfur [μg L^−1^]
Methionine	2.8	0.04	1.4
Methionine sulfone	3.3	0.05	1.7
Cysteine	10.3	0.05	1.5
Cysteic acid	11.3	0.07	2.3
Sulfate	11.7	0.3	9.8

Species-specific isotope dilution using a ^34^S-labeled yeast hydrolysate was chosen for the quantification, as the approach, where an isotope-enriched standard is added online to the sample, does not account for analyte losses during sample preparation.

**Fig. 4:**
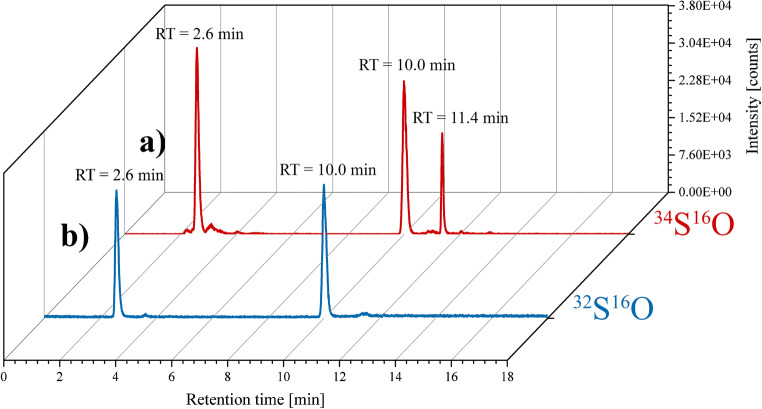
Chromatograms for the measurement of ^34^S-labeled yeast hydrolysate using reverse species-specific isotope dilution and HPLC-ICP-MS/MS analysis. Previously characterized cysteic acid and methionine sulfone standards with natural abundance and 500 μg L^−1^ sulfur concentration (**b**) were spiked to the yeast hydrolysate (**a**). The gradient was slightly modified (ESM Fig. S3) to ensure separation of all the yeast components, which resulted in shifted retention times

For the calculation of the amino acid concentration using isotope dilution, Equation S1 (see ESM) was used. To determine the performance of this method, external calibration with a sulfur standard was also carried out for comparison. In a first step, the ^34^S-labeled yeast hydrolysate was characterized using species-specific reverse isotope dilution (Fig. 4). For this purpose, a mixture with similar concentrations of the yeast hydrolysate (Fig. 4a) and the two amino acid standards methionine sulfone and cysteic acid (Fig. 4b**)** was created. The exact sulfur concentration of the standards was previously quantified by isotope dilution using flow injection ICP-MS as described in the procedure of Fig. 1. The yeast hydrolysate was measured a total of 10 times, and numerous peaks were visible with methionine sulfone, cysteic acid, and sulfate being the most abundant ones.

Both amino acids showed similar concentrations, whereas sulfate was present in lower concentrations (Table 4).

**Table 4:** Mass fractions of the sulfur-containing main components in the ^*34*^S-labeled yeast hydrolysate (*n* = 10) determined by species-specific reverse isotope dilution and HPLC-ICP-MS/MS detection

Substance	Mass fraction [mg g^−1^]
Methionine sulfone	6.65 ± 0.04 (*n* = 10)
Cysteic acid	5.49 ± 0.04 (*n* = 10)
Sulfate	0.42 ± 0.01 (*n* = 10)

The isotopic abundances of sulfur were determined within the yeast (ESM Table S1). No ^32^S peaks were detected, which was beneficial for the IDMS measurements. Only small quantities of ^33^S were visible.

By adding this characterized ^34^S-labeled yeast hydrolysate to the samples, it was possible to accurately quantify the amino acids methionine and cysteine using species-specific isotope dilution and HPLC-ICP-MS/MS. The peptide content was determined by comparing the measured values with the theoretical ones, which were based on the amino acid sequence of the peptides.

### Validation of the developed method

In order to validate the developed method, the standard reference material (SRM) NIST 2389a was subjected to the developed sample preparation procedure. The used SRM is an amino acid mix, containing cysteine and methionine in equimolar concentration (~ 2.5 mmol L^−1^), and the mass fractions were determined by species-specific isotope dilution and external calibration. In the chromatogram, both amino acids showed similar peak areas (ESM Fig. S4). The mass fractions for cysteine and methionine determined by species-specific isotope dilution were in good agreement with the certified values, whereby a recovery of almost 100% was achieved for both amino acids (Table 5). External calibration in contrast provided only recoveries of 70% for both amino acids.

**Table 5:** Measured mass fractions and recoveries for the amino acids in the standard reference material NIST 2389a. These parameters were used to compare the accuracy and precision of external calibration and species-specific isotope dilution. The certified values are shown for comparison

Amino acid	Certified mass fraction [mg g^−1^]	Mass fraction [mg g^−1^] (external calibration)	Recovery [%]	Mass fraction [mg g^−1^] (species-specific ID)	Recovery [%]
Cysteine	0.295 ± 0.013	0.195 ± 0.02 (*n* = 4)	66.12 ± 0.06	0.297 ± 0.01 (*n* = 8)	100.68 ± 1.69
Methionine	0.373 ± 0.011	0.260 ± 0.02 (*n* = 4)	69.73 ± 0.05	0.368 ± 0.01 (*n* = 8)	98.66 ± 3.22

### Measurement of the peptide standards

After validation, the developed method was used to quantify the peptide contents of the protein and peptide standards, as can be seen in Table 6. Since it was not possible to weigh in Aβ accurately, the peptide content here refers to the manufacturer’s specification (r-Peptide) of 1 mg Aβ per vial. In another attempt, where a different vial of Aβ was used, Aβ40 was weighed in completely with the microbalance showing a value of 1.269 mg. Each of the 3 Aβ aliquots was measured 5 times, the protein aliquots 2 times. For external calibration, each fraction was only measured once.

**Table 6:** Peptide content of the amyloid β peptides (40, 42) and protein standards with different grades of purity. The content was determined by comparing the measured value of the sulfur-containing amino acids with the theoretical ones (based on the amino acid sequence). External calibration was used for comparison with species-specific isotope dilution

Peptide content [%]			
Sample	Manufacturer information	External calibration	Species-specific ID
Myoglobin	95–100	53.05 ± 1.02 (*n* = 3)	96.44 ± 3.32 (*n* = 6)
Myoglobin (SDS-PAGE)	≥ 98	59.06 ± 3.75 (*n* = 3)	100.72 ± 2.84 (*n* = 6)
Lysozyme	≥ 90	59.29 ± 5.52 (*n* = 3)	97.44 ± 0.89 (*n* = 6)
Lysozyme (VETRANAL®)	100	61.54 ± 5.33 (*n* = 3)	99.98 ± 1.57 (*n* = 6)
Amyloid β 1-40	98	49.19 ± 3.22 (*n* = 3)	126.78 ± 0.05 (*n* = 15)
Amyloid β 1-42	> 97	19.99 ± 1.71 (*n* = 3)	52.56 ± 0.01 (*n* = 15)

Species-specific isotope dilution resulted in peptide contents similar to the information provided by the manufacturer. The differences between the various degrees of purity were visible, with the analytical versions of the protein standards showing, on average, higher peptide contents. External calibration on the other hand had on average 30–40% of the peptide content missing. Therefore, similar patterns as with NIST 2389a were seen, leading to the conclusion that there were significant losses during sample preparation, which are corrected by using species-specific isotope dilution. The peptide content of Aβ, however, showed a significant deviation from the specifications. Aβ40 showed values that were more than 2.4 times higher than Aβ42. External calibration showed similar ratios between the two peptides. Since the entire content of the vial has been solved and the differences between the individual fractions were small, it can be assumed that there is a problem with the weight specifications of the manufacturer. According to r-Peptide, their Aβ standards are prepared by lyophilizing the corresponding peptide solution into the vial and adding 5–10% more of the product to compensate for losses during resuspension. For this purpose, the peptide solutions are characterized chromatographically using amino acid analysis. This statement could provide a good explanation for the results. With the procedure described by the manufacturer, errors in the determination of the peptide content are possible. In contrast to simple weighing, an amino acid analysis has numerous influencing factors, such as calibration, measuring conditions, or evaluation, resulting in greater uncertainty. In addition to possible losses during lyophilization, the supplement of another 5–10% product makes it impossible to estimate the exact peptide content, so it is misleading to state 1 mg on the analysis certificate.

## Conclusions

This work successfully demonstrated the capabilities of combining species-specific isotope dilution with HPLC-ICP-MS/MS to determine the peptide content and thus the purity of protein standards. This was made possible by a novel ^34^S-labeled yeast hydrolysate, which contains the amino acids methionine sulfone and cysteic acid that are almost completely enriched in ^34^S (98.89%). The optimized HPLC method using a strong anion exchange column provided excellent separation for the sulfur-containing amino acids, as well as their oxidized forms and sulfate. Compared with external calibration, species-specific isotope dilution was able to demonstrate significantly higher precision and accuracy, resulting in recoveries of nearly 100% for the standard reference material (NIST 2389a) and RSD values below 4%. While the purity of the protein standards could be confirmed using this method, the Aβ peptides showed significant deviations, which is most likely due to problems in the manufacturer’s weight specifications. Since even small differences in concentration can be decisive in clinical analysis, species-specific isotope dilution offers a good possibility for the characterization of biological standards in an accurate and traceable manner.

## Supplementary Information

ESM 1 (DOCX 341 kb)

## References

[1] Beltrán-Sánchez H, Soneji S, Crimmins EM (2015). Past, present, and future of healthy life expectancy. Cold Spring Harb Perspect Med.

[2] Calvo-Rodriguez M, Hou SS, Snyder AC, Kharitonova EK, Russ AN, Das S (2020). Increased mitochondrial calcium levels associated with neuronal death in a mouse model of Alzheimer’s disease. Nat Commun.

[3] World Health Organization. The top 10 causes of death. 2018 https://www.who.int/en/news-room/fact-sheets/detail/the-top-10-causes-of-death. Accessed 17 Jun 2020.

[4] Weller J, Budson A (2018). Current understanding of Alzheimer’s disease diagnosis and treatment. F1000Res.

[5] Bateman RJ, Xiong C, Benzinger TLS, Fagan AM, Goate A, Fox NC (2012). Clinical and biomarker changes in dominantly inherited Alzheimer’s disease. N Engl J Med.

[6] Association As (2015). 2015 Alzheimer’s disease facts and figures. Alzheimers Dement.

[7] Perl DP (2010). Neuropathology of Alzheimer’s disease. Mt Sinai J Med.

[8] Goldsworthy MR, Vallence A-M (2013). The role of β-amyloid in alzheimer's disease-related neurodegeneration. J Neurosci.

[9] de Paula V, Guimarães FM, Diniz BS, Forlenza OV (2009). Neurobiological pathways to Alzheimer’s disease: amyloid-beta, TAU protein or both?. Dement Neuropsychol.

[10] O'Brien RJ, Wong PC (2011). Amyloid precursor protein processing and Alzheimer’s disease. Annu Rev Neurosci.

[11] Das B, Dasgupta S, Ray S (2019). Potential therapeutic roles of retinoids for prevention of neuroinflammation and neurodegeneration in Alzheimer’s disease. Neural Regen Res.

[12] Blennow K, Zetterberg H (2018). Biomarkers for Alzheimer’s disease: current status and prospects for the future. J Intern Med.

[13] Sharma N, Singh AN (2016). Exploring biomarkers for Alzheimer’s disease. J Clin Diagn Res.

[14] Verwey NA, Veerhuis R, Twaalfhoven HAM, Wouters D, Hoozemans JJM, Bollen YJM (2009). Quantification of amyloid-beta 40 in cerebrospinal fluid. J Immunol Methods.

[15] Schoonenboom NS, Mulder C, Vanderstichele H, Pijnenburg YA, Van Kamp GJ, Scheltens P (2005). Differences and similarities between two frequently used assays for amyloid β 42 in cerebrospinal fluid. Clin Chem.

[16] Bjerke M, Portelius E, Minthon L, Wallin A, Anckarsäter H, Anckarsäter R (2010). Confounding factors influencing amyloid beta concentration in cerebrospinal fluid. Int J Alzheimers Dis.

[17] Ellis TA, Li J, LeBlond D, Waring JF (2012). The relationship between different assays for detection and quantification of amyloid beta 42 in human cerebrospinal fluid. Int J Alzheimers Dis.

[18] Niemantsverdriet E, Ottoy J, Somers C, De Roeck E, Struyfs H, Soetewey F (2017). The cerebrospinal fluid Aβ1-42/Aβ1-40 ratio improves concordance with amyloid-PET for diagnosing Alzheimer’s disease in a clinical setting. J Alzheimers Dis.

[19] Kessler A (2016). Mass spectrometry – a key technique for traceability in clinical chemistry. TrAC Trends Anal Chem.

[20] Hermann G, Møller LH, Gammelgaard B, Hohlweg J, Mattanovich D, Hann S (2016). In vivo synthesized 34S enriched amino acid standards for species specific isotope dilution of proteins. J Anal At Spectrom.

[21] JCGM 200:2012(E/F). International vocabulary of metrology – basic and general concepts and associated terms (VIM). Joint Committee for Guides in Metrology. 2012 https://www.bipm.org/utils/common/documents/jcgm/JCGM_200_2012.pdf. Accessed 10 Mar 2021.

[22] ISO 17511:2020. In vitro diagnostic medical devices — requirements for establishing metrological traceability of values assigned to calibrators, trueness control materials and human samples. 2020 International Organization for Standardization, Geneva. https://www.iso.org/obp/ui/#iso:std:iso:17511:ed-2:v1:en. Accessed 10 Mar 2021.

[23] Barwick VJ, Prichard E. Eurachem guide: terminology in analytical measurements – introduction to VIM3. 2011 https://www.eurachem.org/images/stories/Guides/pdf/TAM_2011_Final_web.pdf. Accessed 10 Mar 2021.

[24] Swart C (2013). Metrology for metalloproteins—where are we now, where are we heading?. Anal Bioanal Chem.

[25] Bradford MM (1976). A rapid and sensitive method for the quantitation of microgram quantities of protein utilizing the principle of protein-dye binding. Anal Biochem.

[26] Crabb JW, West KA, Dodson WS, Hulmes JD (2001). Amino acid analysis. Curr Protoc Protein Sci.

[27] Hussain MT, Forbes N, Perrie Y (2019). Comparative analysis of protein quantification methods for the rapid determination of protein loading in liposomal formulations. Pharmaceutics..

[28] Oe T, Ackermann BL, Inoue K, Berna MJ, Garner CO, Gelfanova V (2006). Quantitative analysis of amyloid beta peptides in cerebrospinal fluid of Alzheimer's disease patients by immunoaffinity purification and stable isotope dilution liquid chromatography/negative electrospray ionization tandem mass spectrometry. Rapid Commun Mass Spectrom.

[29] Kaneko N, Yamamoto R, Sato T-A, Tanaka K (2014). Identification and quantification of amyloid beta-related peptides in human plasma using matrix-assisted laser desorption/ionization time-of-flight mass spectrometry. Proc Jpn Acad Ser B Phys Biol Sci.

[30] Fernández SD, Sugishama N, Encinar JR, Sanz-Medel A (2012). Triple quad ICPMS (ICPQQQ) as a New Tool For Absolute Quantitative Proteomics And Phosphoproteomics. Anal Chem.

[31] Lee H-S, Kim SH, Jeong J-S, Lee Y-M, Yim Y-H (2015). Sulfur-based absolute quantification of proteins using isotope dilution inductively coupled plasma mass spectrometry. Metrologia..

[32] Giné M, Packer AP (2010). Online isotope dilution and inductively coupled plasma mass spectrometry: from elemental to species quantification. J Braz Chem Soc..

[33] Feng L, Huo Z, Xiong J, Li H (2020). Certification of amyloid-beta (Aβ) certified reference materials by amino acid-based isotope dilution high-performance liquid chromatography mass spectrometry and sulfur-based high-performance liquid chromatography isotope dilution inductively coupled plasma mass spectrometry. Anal Chem.

[34] Pedrero Z, Ruiz Encinar J, Madrid Y, Cámara C (2007). Application of species-specific isotope dilution analysis to the correction for selenomethionine oxidation in Se-enriched yeast sample extracts during storage. J Anal At Spectrom.

[35] Deitrich CL, Braukmann S, Raab A, Munro C, Pioselli B, Krupp EM (2010). Absolute quantification of superoxide dismutase (SOD) using species-specific isotope dilution analysis. Anal Bioanal Chem.

[36] Inagaki K, Takatsu A, Watanabe T, Aoyagi Y, Okamoto K (2003). Species-specific isotope dilution analysis of mono-, di, and tri-butyltin compounds in sediment using gas chromatography-inductively coupled plasma mass spectrometry with synthesized 118Sn-enriched butyltins. Analyst..

[37] Hermann G, Schwaiger M, Volejnik P, Koellensperger G (2018). 13C-labelled yeast as internal standard for LC–MS/MS and LC high resolution MS based amino acid quantification in human plasma. J Pharm Biomed Anal.

[38] Rampler E, Dalik T, Stingeder G, Hann S, Koellensperger G (2012). Sulfur containing amino acids – challenge of accurate quantification. J Anal At Spectrom.

[39] Galilea San Blas O, Moreno Sanz F, Herrero Espílez P, Prieto García B, Álvarez Menéndez FV, Marchante-Gayón JM (2016). Determination of free methionine in human blood plasma by species-specific isotope dilution HPLC-ICP-MS using 34S-labelled methionine. J Anal At Spectrom.

