# Supplementary material for: Accurate characterization of β-amyloid (Aβ40, Aβ42) standards using species-specific isotope dilution by means of HPLC-ICP-MS/MS
Source: Anal Bioanal Chem. 2021 Aug 6;414(1):639–48. doi: 10.1007/s00216-021-03571-6 (PMC8748378; doi:10.1007/s00216-021-03571-6)
Supplement: Supplementary file 1 — (DOCX 341 kb) [file 216_2021_3571_MOESM1_ESM.docx]

**Supplementary Information**


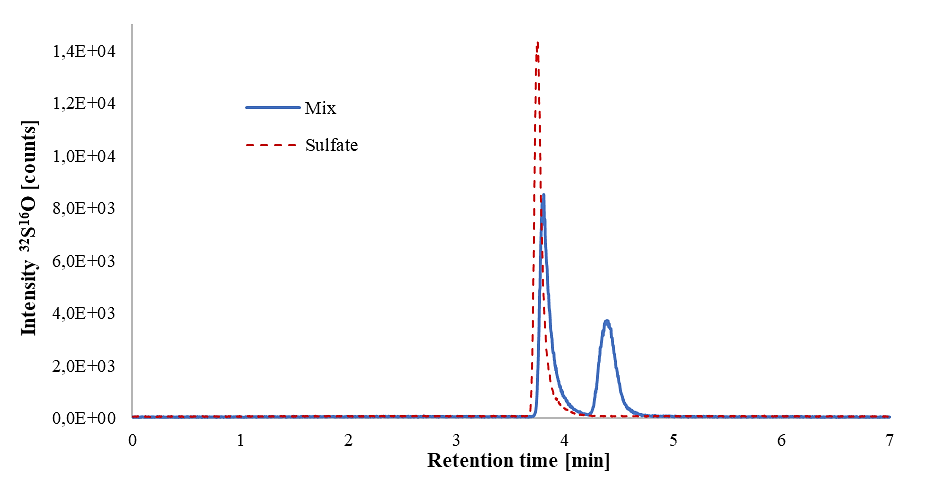


**Figure S1:** Chromatogram of a 2 mg L^-1^ amino acid mixture containing methionine, cysteine and their oxidized forms. A 500 µg L^-1^ sulfate standard is seen in the background overlapping with the cysteine peak due to similar retention times. The sulfur trace was monitored by HPLC-ICP-MS/MS using oxygen as reaction gas.


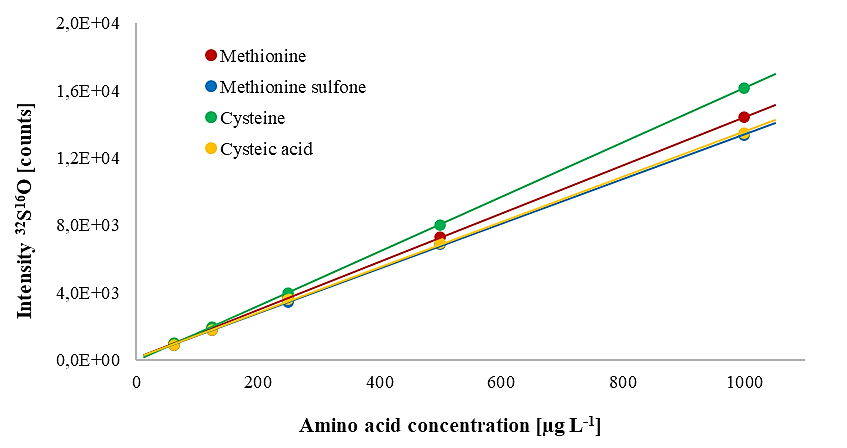


**Figure S2:** Calibration curves of the different amino acids in a concentration range between 50-1000 µg L^-1^. The sensitivity was dependent on the sulfur content of the substance. Sulfur was measured using HPLC-ICP-MS/MS with oxygen as reaction gas.


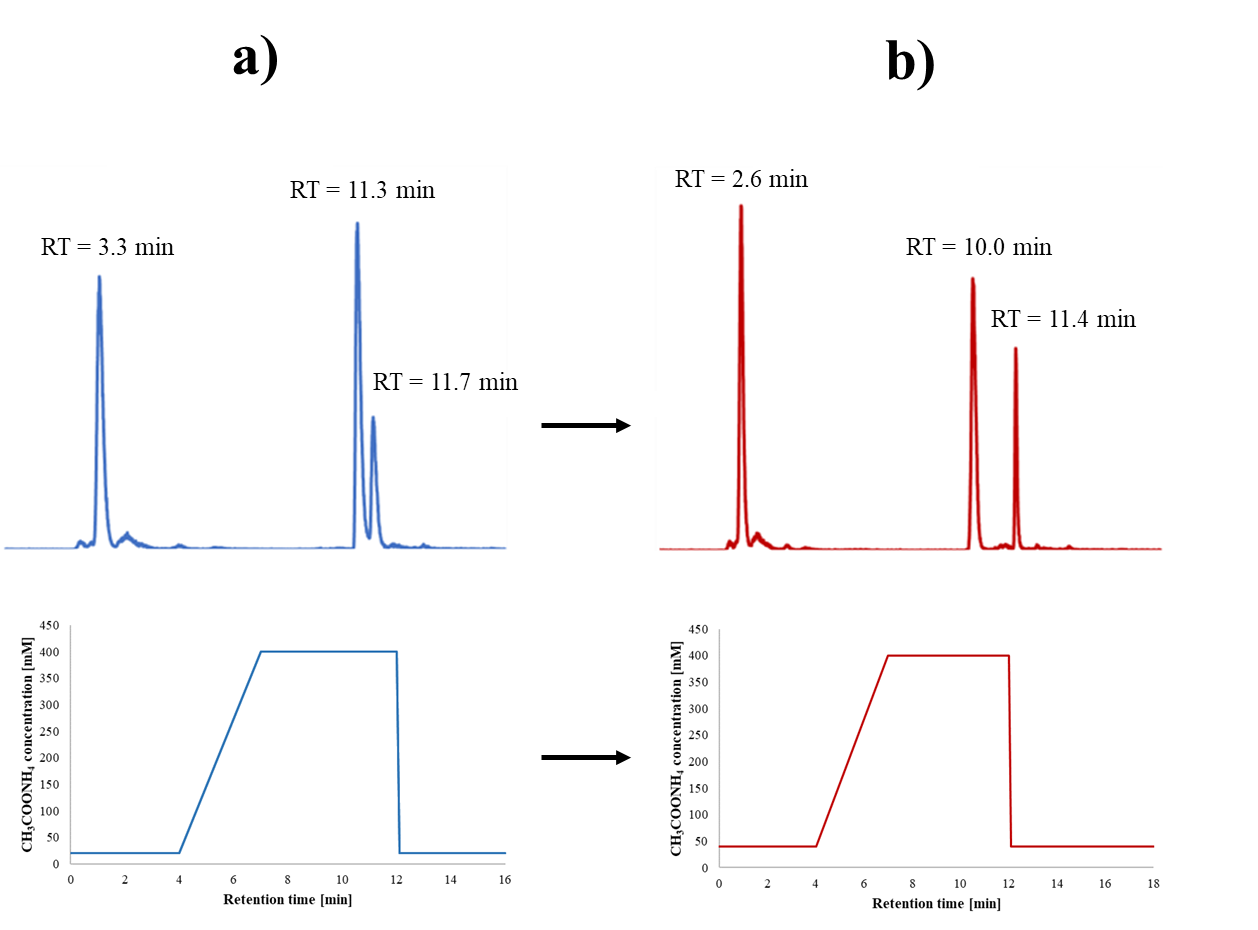


**Figure S3:** Chromatograms for the measurement of ^34^S-labelled yeast hydrolysate using HPLC-ICP-MS/MS analysis. Due to the increased number of components in yeast, there was the possibility for peak overlap with the original gradient (a), which is why the gradient was slightly modified for better separation. The starting concentration was changed to 40 mM CH_3_COONH_4_ and the measurement time was extended by 2 minutes, resulting in a better visibility for the less abundant peaks (b).

**
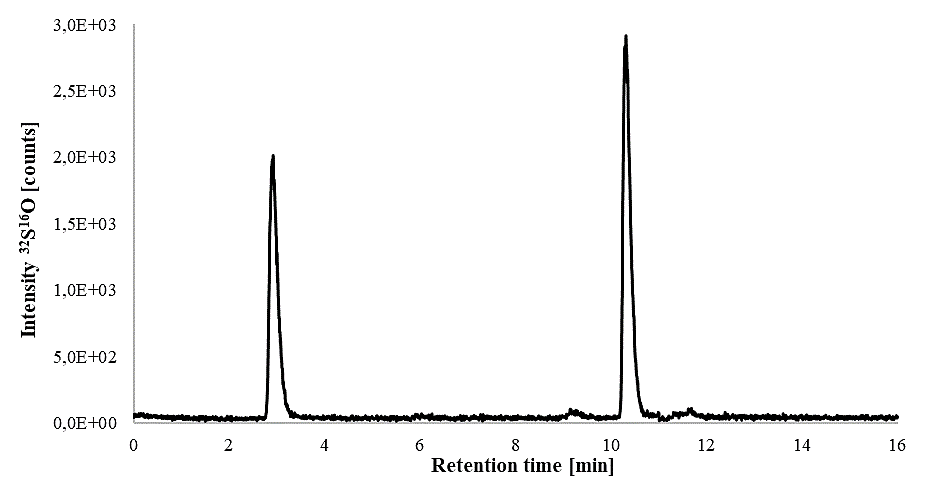
**

**Figure S4:** Chromatogram of the NIST 2389a standard reference material after being exposed to hydrolysis and oxidation. Prior to analysis the SRM was diluted 1:200 resulting in a concentration of approximately 1.5 mg L^-1^ for methionine and 2 mg L^-1^ for cysteine.

**Table S1:** Isotopic composition of the ^34^S-labelled yeast hydrolysate. The signals for ^32^S and ^36^S were under the limit of detection.

| Isotopic abundance [%] | | | |
| --- | --- | --- | --- |
| ^32^S | **^33^S** | **^34^S** | **^36^S** |
| < LOD | 1.11 | 98.89 | < LOD |

**Equation S1:** Determination of the compound concentration using isotope dilution. Here, x relates to the sample with natural isotopic composition, s to the isotopically enriched spike and b is the blend of both. R represents the ratio between the measured isotopes (^32^S/^34^S) and f the abundance of a single isotope.

$$C_{x}=\frac{C_{s}\cdot m_{s}}{m_{x}}\cdot\frac{R_{s}-R_{b}}{R_{b}-R_{x}}\cdot\frac{f_{Bs}}{f_{Bx}}$$
